# Idiopathic atrial fibrillation patients rapidly outgrow their low thromboembolic risk: a 10-year follow-up study

**DOI:** 10.1007/s12471-019-1272-z

**Published:** 2019-04-05

**Authors:** B. Weijs, E. A. M. P. Dudink, C. B. de Vos, I. Limantoro, R. G. Tieleman, R. Pisters, E. C. Cheriex, J. G. L. M. Luermans, H. J. G. M. Crijns

**Affiliations:** 1 Maastricht University Medical Center and Cardiovascular Research Institute Maastricht, Maastricht, The Netherlands; 2 Laurentius Hospital, Roermond, The Netherlands; 3 Martini Hospital, Groningen, The Netherlands; 4 Rijnstate Hospital, Arnhem, The Netherlands

**Keywords:** Atrial fibrillation, Lone, Idiopathic, Anticoagulation, CHA_2_DS_2_-VASc, Follow-up, Stroke, Thromboembolism

## Abstract

**Background:**

Healthy atrial fibrillation (AF) patients will eventually outgrow their low thromboembolic risk. The purpose of this study is to compare the development of cardiovascular disease in healthy AF patients as compared to healthy sinus rhythm patients and to assess appropriate anticoagulation treatment.

**Methods:**

Forty-one idiopathic paroxysmal AF patients (56 ± 10 years, 66% male) were compared with 45 healthy sinus rhythm patients. Patients were free of hypertension, antihypertensive and antiarrhythmic drugs, diabetes, congestive heart failure, coronary artery or peripheral vascular disease, previous stroke, thyroid, pulmonary and renal disease, and structural abnormalities on echocardiography.

**Results:**

Baseline characteristics and echocardiographic parameters were the same in both groups. During 10.7 ± 1.6 years, cardiovascular disease and all-cause death developed significantly more often in AF patients as compared to controls (63% vs 31%, log rank *p* < 0.001). Even after the initial 5 years of follow-up, survival curves show divergent patterns (log rank *p* = 0.006). Mean duration to reach a CHA_2_DS_2_-VASc score > 1 among AF patients was 5.1 ± 3.0 years. Five of 24 (21%) patients with CHA_2_DS_2_-VASc > 1 did not receive oral anticoagulation therapy at follow-up. Mean duration of over- or undertreatment with oral anticoagulation in patients with CHA_2_DS_2_-VASc > 1 was 5 ± 3.0 years.

**Conclusion:**

The majority of recently diagnosed healthy AF patients develop cardiovascular diseases with a consequent change in thromboembolic risk profile within a short time frame. A comprehensive follow-up of this patient category is necessary to avoid over- and undertreatment with anticoagulants.

## What’s new?

- The majority of recently diagnosed healthy patients with atrial fibrillation (AF) develop cardiovascular diseases, with a consequent change in thromboembolic risk, within a short time frame.
- The average time to develop a CHA_2_DS_2_-VASc score > 1 was roughly only 4 years.
- Despite an increase in the CHA_2_DS_2_-VASc score during follow-up, many initally low-risk patients did not ultimately receive adequate (N)OAC treatment.
- The mean duration of untreated thromboembolic risk among inadequately treated patients was 5.1 ± 2.8 years.
- A comprehensive follow-up of AF patients with an initially low thromboembolic risk is necessary to prevent cardiovascular disease progression and to avoid undertreatment with anticoagulant therapy.

## Introduction

During the past few years, physicians have made a great effort to let the medical world understand the importance of adequate recognition of atrial fibrillation (AF). Since this encourages opportunistic screening for the arrhythmia, the development of hand-held, easy-to-use devices to detect AF is progressing rapidly [1, 2]. We currently face a huge number of newly detected AF patients. A large proportion of these newly detected AF patients might be young, otherwise healthy and asymptomatic.

Although there is conflicting evidence in the literature concerning the prognosis and risk for development of comorbidities among these healthy AF patients, one could assume that the ‘idiopathic’ type of the arrhythmia may act as a whistle-blower of subclinical heart disease [3, 4].

Current guidelines on AF management state that the specialist caring for the AF patient should not only perform the baseline assessment and institute the appropriate treatment, but should also suggest a structural plan for follow-up. However, the guidelines do not provide a plan for how to do so. It might be challenging in the young and healthy AF patient, with adequate rhythm control and sporadic or asymptomatic paroxysms, to devise an appropriate time frame for follow-up visits. Eventually, these patients will outgrow their low thromboembolic risk. The question remains when this will take place, whether the patient or their physician will be aware of that, and whether antithrombotic medication will be adjusted accordingly.

We found it useful for clinical practice to study the development of cardiovascular disease (CVD), the increase of thromboembolic risk and the prescription of antithrombotic therapy over time in recently diagnosed healthy AF patients with an initially low CHA_2_DS_2_-VASc risk score in common daily practice (not specialised AF care).

## Methods

### Study population

The studied population has been described previously [3]. In brief, we studied 41 consecutive idiopathic paroxysmal AF patients and 45 healthy sinus rhythm (SR) control patients who were referred to the outpatient clinic of one of the authors between 2004 and 2007 for a standard transthoracic echocardiographic examination (in the work-up for AF or for cardiovascular screening purposes in the SR control population). Individual AF treatment and follow-up/discharge was left to the discretion of the treating physician. The study was approved by the local Ethics Committee and complied with the Declaration of Helsinki. Informed consent of all patients was obtained. In the current report we extended the total follow-up duration and focused on the anticoagulation strategy.

Idiopathic AF and healthy SR were strictly defined as the absence of any CVD including hypertension, diabetes, or hypercholesterolaemia. In addition, no coronary artery disease (from absence of typical exercise-related angina pectoris and beyond), no peripheral vascular disease, no congestive heart failure (clinical signs or symptoms, ejection fraction > 50%, normal diastolic function), no previous stroke, no thyroid or pulmonary disease, preserved renal function (MDRD > 60 ml/min/1.73m^2^), no malignancy, and no evidence for structural CVD on echocardiography. Female gender was not included in CHA_2_DS_2_-VASc in the absence of other stroke risk factors [5, 6].

The combined endpoint was defined as all emerging CVDs, including all-cause death, thromboembolic complications, congestive heart failure (left ventricular ejection fraction, LVEF, < 45%, admission), significant coronary artery disease, diabetes mellitus, and new onset hypertension (need for antihypertensive drugs, or development of hypertrophy > 10 mm). Major adverse cardiac and cerebrovascular events (MACCE) were defined as cardiovascular death, stroke, myocardial infarction or repeated revascularisation, and the occurrence of acute heart failure.

### Data collection

Patient characteristics at the time of echocardiography and during follow-up were obtained. At the end of follow-up, general practitioners and anticoagulation clinics were contacted in order to obtain additional follow-up data.

### Statistical analysis

Statistical analysis was performed using SPSS statistical software (IBM SPSS statistics version 23.0, IBM Corporation, Armonk, NY, USA). Categorical variables are reported as number of patients and percentage and were compared using chi-square testing. Continuous variables are presented as mean ± standard deviation or median (interquartile range) and were compared with an independent *t*-test (two-tailed) for normally distributed continuous variables and Mann–Whitney test (two-tailed) for all not normally distributed variables. All parameters showing a significant univariable relation with the occurrence of CVD during follow-up using Cox regression were included as covariates in a multivariate Cox regression model. Results were checked for colinearity and interaction among covariates. Proportional hazards were checked. Manual backwards selection was used to construct the final models (retention level set at 0.10). Hazard ratios (HR) and 95% confidence intervals (CI) were calculated. A *p*-value of 0.05 was considered statistically significant.

## Results

### Cardiovascular disease during follow-up

Baseline characteristics were the same in AF cases and controls (Tab. 1; [3]).

**Table 1:** Baseline characteristics

	sinus rhythm (*n* = 45)	atrial fibrillation (*n* = 41)	*p*-value
*demographics*			
age (years), median (IQR)	57 (17)	58 (17)	0.465
male	23 (51)	27 (66)	0.194
body mass index (kg/m^2^)	26 (4)	27 (4)	0.348
AF history (months)	–	1 (10)	
*medication*			
oral anticoagulation	0	9 (22)	<0.001
aspirin	5 (11)	18 (44)	0.001
beta-blocker	7 (16)	13 (32)	0.124
*echocardiography*			
aorta diameter (mm)	33 (3)	34 (4)	0.056
left atrial diameter (mm)	38 (4)	39 (5)	0.254
left ventricular end-diastolic diameter (mm)	48 (4)	49 (4)	0.191
left ventricular end-systolic diameter (mm)	32 (4)	33 (3)	0.261
interventricular septum width (mm)	8.7 (0.9)	8.6 (0.8)	0.763
posterior wall width (mm)	8.4 (0.8)	8.5 (0.7)	0.693
left ventricular ejection fraction (%)	62 (5)	62 (4)	0.976

Data are presented as mean (±SD) or *n* (%), unless specified otherwise

*AF* atrial fibrillation

Tab. 2 shows follow-up characteristics. Follow-up was incomplete in 4 patients (mean follow-up in these patients was 4.8 ± 1.1 years). During a mean period of 10.7 ± 1.6 years from baseline, patients with AF died or developed CVD significantly more often than SR controls (63% vs 31%, log rank *p* < 0.001). Even after the initial 5 years of follow-up, survival curves show divergent patterns (log rank *p* = 0.006). Log rank *p*-values for true MACCE (all-cause death excluded) are 0.005 for complete follow-up and 0.034 after the initial 5 years of follow-up. The Kaplan-Meier curve with cumulative incidence of CVD during the first 5 years of follow-up was published previously [3].

**Table 2:** Atrial fibrillation progression, cardiovascular disease and medication during follow-up

	sinus rhythm (*n* = 45)	atrial fibrillation (*n* = 41)	*p*-value
*demographics*			
age (years) at end of follow-up, median (IQR)	66 (18)	67 (19)	0.243
clinical progression of atrial fibrillation	4 (8.9)	19 (46.3)	<0.001
visits to cardiac emergency department	0.1 (0)	1.7 (2.8)	<0.001
death	2 (4.4)	5 (12.2%)	0.189
malignancy	2 (4.4)	3 (7.3)	
pneumosepsis	0	1 (2.4)	
out-of-hospital cardiac arrest	0	1 (2.4)	
*cardiovascular (related) disease during follow-up* ^a^	14 (31)	22 (54)	0.034
cerebrovascular accident	1 (2.2)	2 (4.9)	0.503
significant coronary artery disease	1 (2.2)	5 (12.2)	0.070
congestive heart failure	1 (2.2)	5 (12.2)	0.070
arterial hypertension	10 (22.2)	16 (39)	0.090
venous thromboembolism	2 (4.4)	0	0.172
diabetes mellitus	5 (11.1)	3 (7.3)	0.545
major/minor bleeding complications	0	0	1.0
total number of patients with cardiovascular disease and/or death^b^	14 (31)	26 (63)	0.003
*medication*			
vitamin K antagonist	2 (4.5)	18 (43.9)	<0.001
direct oral anticoagulant	1 (2.3)	7 (17.1)	0.020
aspirin	7 (15.9)	7 (17.1)	0.885
beta-blocker	4 (9.1)	12 (29.3)	0.017
sotalol	2 (4.5)	8 (19.5)	0.032
flecainide	0	8 (19.5)	0.002
amiodarone	0	5 (12.2)	0.017
anti-hypertensive drug use^c^	12 (26.7)	22 (53.7)	0.011
statin	9 (20.5)	11 (26.8)	0.489

Data are presented as mean (±SD) or *n* (%), unless specified otherwise

^a^The tabulations of cardiovascular diseases during follow-up include the first event for each patient

^b^Some patients developed cardiovascular disease and died later during follow-up. Statistical tests were performed based on first event only

^c^ACE inhibitor, angiotensin receptor blocker, diuretics, calcium channel blocker

Although not statistically significant, patients with AF were younger than controls at the time of their first cardiovascular event or associated disease (60.5 ± 9 years vs 65.0 ± 0 7 years, *p* = 0.103). Cerebrovascular accidents occurred in two AF patients (1 ischaemic stroke, 1 transient ischaemic attack), and one SR patient was diagnosed with AF during follow-up and experienced two transient ischaemic attacks thereafter.

Multivariable Cox regression analysis revealed that baseline posterior wall width (8.3 ± 0.7 vs 8.7 ± 0.8 mm, *p* = 0.013. HR 1.9; 1.2–2.9, *p* = 0.003), a history of AF (38% vs 64%, *p* = 0.019. HR 2.6; 1.3–4.9, *p* = 0.005), and age (50 ± 13 vs 57 ± 8 years, *p* = 0.012. HR 1.0; 1.0–1.1, *p* = 0.018) were independent significant predictors of CVD development.

### Anticoagulation

During follow-up a total of 4 SR patients developed AF. As a result, there were 45 patients with a diagnosis of AF to study the prescription of antithrombotic therapy at the end of follow-up. Two female patients (aged 65 and 67 years) had a CHA_2_DS_2_-VASc score of 2 at baseline; these patients were excluded from calculation of duration to reach a CHA_2_DS_2_-VASc score of 2.

**Fig. 1:**
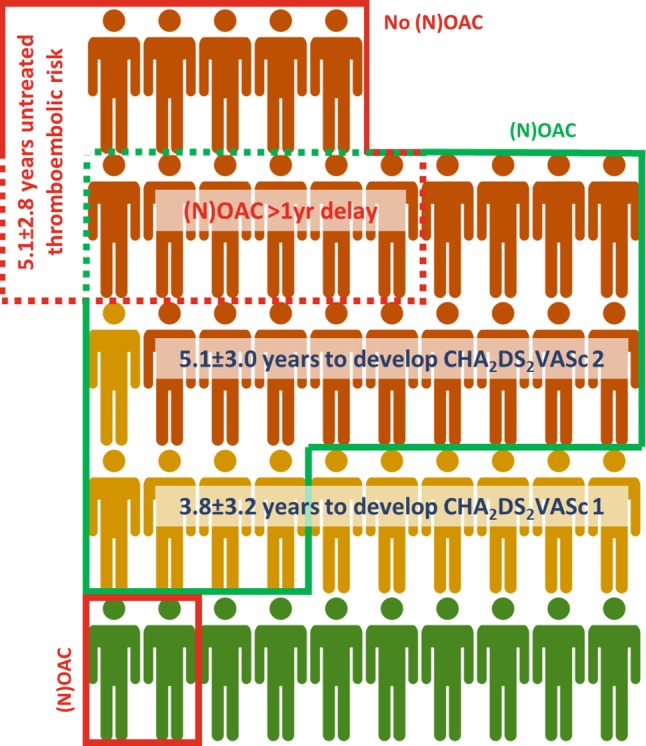
Development of increased CHA_2_DS_2_-VASc score, as well as prescription rates of (novel) oral anticoagulants, (N)OAC, in idiopathic atrial fibrillation patients during 10 years of follow-up. Green icons: CHA_2_DS_2_-VASc score 0; yellow icons: CHA_2_DS_2_-VASc score 1; orange icons: CHA_2_DS_2_-VASc score >1. Green line: adequate OAC use; red line: inadequate OAC use

Among 45 AF patients, a total of 35 (78%) developed a CHA_2_DS_2_-VASc score of 1 or higher, of which 24 (53%) developed a CHA_2_DS_2_-VASc of 2 or higher. A total of 10 (22%) patients retained their low thromboembolic risk (CHA_2_DS_2_-VASc 0). Fig. 1 shows characteristics regarding the development and distribution of increased CHA_2_DS_2_-VASc score during follow-up, as well as the prescription of (novel) oral anticoagulants, (N)OAC.

**Fig. 2:**
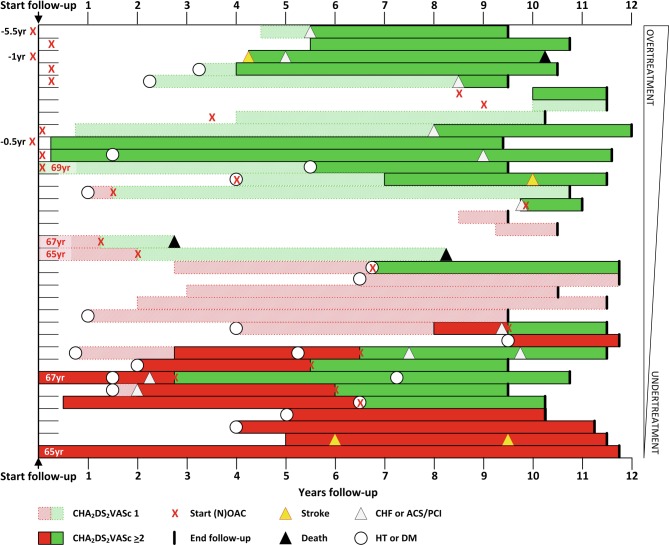
Time to increased CHA_2_DS_2_-VASc score. Start of oral anticoagulation (OAC) therapy and occurrence of major adverse cardiac and cerebrovascular events in 35 of 45 individual atrial fibrillation patients (CHA_2_DS_2_-VASc score remained at 0 in 10 patients during follow-up, 3 patients had already been incorrectly treated with OAC prior to initial visit, *green/red* for adequate/inadequate OAC at time of increased thromboembolic risk). Patients are ranked according to total duration of over-/undertreatment with OAC therapy. *ACS* acute coronary syndrome, *CHF* congestive heart failure, *DM* diabetes mellitus, *HT* hypertension, *PCI* percutaneous coronary intervention, *yr* years

A total of 24 of 35 patients with CHA_2_DS_2_-VASc ≥ 1 received (N)OAC. This treatment was started within 12 months prior to or after an increased thromboembolic risk score in only 8 patients, and 5 of these patients had a CHA_2_DS_2_-VASc score of 1. In 11 (31%) of 35 patients, (N)OAC were started while CHA_2_DS_2_-VASc was still 0 at that timepoint. Overall the mean duration of over- or undertreatment with oral anticoagulation was 5.0 years (±3.0) when CHA_2_DS_2_-VASc > 1 was used as treatment cut-off and 4.2 years (±3.0) when CHA_2_DS_2_-VASc = 1 was used. Fig. 2 shows the duration of increased thromboembolic risk score, start of anticoagulation therapy and the occurrence of MACCE for individual patients with CHA_2_DS_2_-VASc ≥ 1.

## Discussion

Focussing on the early detection of AF during recent years seems to be paying off as the incidence and awareness of AF increase. Opportunistic screening as well as adequate recognition of the arrhythmia leads to an increased detection of AF early in its disease process. It is currently unclear how we should organise the follow-up of this specific patient population with an initially low thromboembolic stroke risk. This study presents a detailed description of the long-term follow-up and real-world treatment of such a presumed healthy patient group.

### Importance of masked or prehypertension

More than half of the ‘healthy’ AF patients developed a cardiovascular condition during 10 years of follow-up. In fact, the average time to develop a CHA_2_DS_2_-VASc score ≥ 1 was roughly only 4 years.

Although patients with hypertension or left ventricular hypertrophy were excluded, posterior wall width at baseline (8.3 vs 8.7 mm) was a predictor of CVD. This suggests the presence of masked or prehypertension in patients participating in this study, and that even normal wall sizes show a variation that potentially represents preclinical disease once wall width is found to be at the high end of normal. This emphasises the importance of regular 24-h ambulatory blood pressure monitoring and adequate treatment of early hypertension in patients with idiopathic AF, since it relates to their vascular prognosis [7].

### Thromboembolic versus (iatrogenic) bleeding risk in young and healthy AF patients

The patients were treated according to common daily practice (not specialised AF care). Patient dismissal, follow-up and (anticoagulant) treatment were left to the discretion of the treating physician. Interestingly, vitamin K antagonists (OAC) or NOAC were often (34%) prescribed when a patient still had CHA_2_DS_2_-VASc 0 (i. e. preceding elective electrical cardioversion) and not stopped subsequently. Further, 5 (21%) patients with CHA_2_DS_2_-VASc ≥ 2 were not receiving (N)OAC at the end of the follow-up period. An explanation might be that some patients did not have an indication for anticoagulation at baseline according to previous risk schemes (Birmingham risk schema and CHADS_2_) and that patients were discharged from regular follow-up due to their healthy profile. These numbers are, however, comparable with the recent results of GARFIELD-AF and GLORIA-AF and confirm that there is still much to be gained regarding guideline-adherent AF management in common daily practice [8–11].

Anticoagulation therapy reduces morbidity and mortality in patients with AF who are at risk for thromboembolism. Since NOAC are associated with low bleeding risk and thromboembolic risk increases within a short time frame in recently diagnosed AF patients with a low CHA_2_DS_2_-VASc score, it is worth considering starting anticoagulation in these patients irrespective of their thromboembolic risk score. Based on currently available data, the net clinical benefit of such a treatment approach is negative due to increased bleeding rates and low baseline thromboembolic rates (stroke risk 0.3–0.7% per year in CHA_2_DS_2_-VASc score 0 vs NOAC major bleeding rates: 1.4–3.6%) [12–15]. It must be stated that the average age in these CHA_2_DS_2_-VASc score 0 populations is somewhat lower than the average age in our study. A randomised trial exploring the potential benefit of (low-dose?)—NOAC in CHA_2_DS_2_-VASc score 0 AF patients aged 50–64 years would certainly add to this consideration but would require a large study population [16].

A recently proposed novel score predicting net clinical outcome may improve risk prediction in this (especially low-risk) population, and could help guide the selection of anticoagulants [17]. Moreover, there are currently many efforts to redefine AF types into biologically plausible subtypes and to improve identification of those patients who currently have only ‘subclinical’ stroke risk factors (prehypertension, prediabetes, age 50–64 years), yet harbour a considerable stroke risk—for example through imaging or biomarker studies [18, 19].

### A dedicated nurse-led ‘smart’ AF service and follow-up plan

Physicians should be encouraged to give high priority to detecting early stages of underlying CVD and to set up a solid individual follow-up plan. Review by a specialised AF service is necessary and follow-up may very well be undertaken in primary care or ideally in a nurse-led AF clinic, as these have been shown to improve survival and quality of life and are a cost-effective management strategy for patients with AF [20, 21]. The implementation of E‑health may actively involve patients in their own care and improve guideline-adherent AF management. At least the individual low-risk AF patient should be carefully instructed to contact their AF specialist in case of disease progression, age > 65 years, or the emergence of hypertension or diabetes mellitus in order to adjust antithrombotic therapy in a timely fashion.

In current practice, many of the patients described in this study are discharged from follow-up. We would like to emphasise the need to continue assessing their stroke risk, as CVD may soon emerge. Annual visits may be too large a burden on healthcare systems, with increasingly limited resources. Therefore, according to the findings in the present study we chose a 2- to 3‑year interval, leaving more frequent visits to the treating physicians’ discretion.

### Limitations

The study results were obtained in a relatively small and—because of our research focus—highly selected population. Nevertheless, it seems reasonable to state that a substantial number of AF patients originally diagnosed with a low thromboembolic risk develop CVD during short-term follow-up. Since SR patients were referred for cardiovascular screening purposes rather than AF, referral bias may have played a role but would—if anything—have led to a higher incidence of events in the control population rather than the reverse. Although we meticulously tried to rule out hypertension, specific cases of masked hypertension may have been missed, since 24-h ambulatory blood pressure monitoring was not performed.

## Conclusion

The majority of recently diagnosed AF patients with low tromboembolic risk develop CVD with a consequent change in thromboembolic risk profile, within a short time frame. A comprehensive follow-up of this specific patient category is necessary to prevent CVD progression and to avoid over- and undertreatment with anticoagulants.
